# Chats about Medical Museums

**Published:** 1887-06-11

**Authors:** 


### Chats about Medical Museums.

By One of the Crowd.

(*Continued from p. 146, vol. ii.*)

### VII.—King's College.

There are two museums at King's College, one devoted to non-medical branches of physical science, and the other to anatomy, pathology, and materia medica, to which, however, are added, for the sake of the general curriculum of the college, collections illustrative of geology, and to some extent of botany. It is of course only with the "medical" department that this article has to do.

The building in which this is found is, like most of the others that have been alluded to in these pages, a spacious hall with galleries round; but it is to a great extent spoiled by the ponderous beams which constitute the greater part of its roof, and which render the skylights embedded among them insufficient to light satisfactorily the extensive collections below. The place is somewhat gloomy in consequence, and the contents of it are just now undergoing rearrangement in many important respects,—the curator, with a zealous devotion characteristic of the best of our younger scientific men, having taken in hand all sorts of schemes for increasing the practical usefulness of the institution. What was said in a previous article respecting the shortcomings of catalogues in some of the museums does not apply to this one. There is no printed catalogue here, but there is a manuscript one, thoroughly well posted up to date; and the various objects in the museum are, moreover, most of them provided with descriptive labels. The curator, however, is doing away with these labels and resorting to numbers only. This is admittedly less satisfactory to the non-professional visitor, but it is found to be better for students, who are, it is said, not so well able to test their knowledge of preparations while they have before their eyes explanatory tablets.

The "popular" element is not very apparent at first sight in this institution. Glass cupboards full of skeletons of kangaroos and alligators, or of men and monkeys, look exceedingly "dry" in a double sense; and long shelves full of incomprehensible bottles offer to the casual observer little that his uninstructed intellect can take in in the way of idea. The dissected body of a snail is to the anatomist no doubt an exceedingly interesting object, but it is not a particularly instructive one to anybody but the anatomist; and to find oneself *vis-à-vis* with the actual head of a patient who has died from a particularly virulent form of small-pox, is, it must be confessed, more sensational than interesting. Those, by the way, who are so vehement in their insistence on the horrors of disorders which are said in exceedingly rare cases to follow vaccination, might find two or three models and preparations in this museum very instructive. Many good people, in their determination to run not the remotest risk of mischief from vaccination, appear to forget altogether that there are any terrors in small-pox. A sight of these models would be very useful to them.

One of the bottles in this museum shows what probably is the smallest and most rudimentary human figure the eye ever looked upon—a veritable baby perhaps as big as a small horse-bean. In another is a portion of a foot, showing the remarkable manner in which the toe-nails grow when they are uncut, and the foot is not used. Some of the Orientals let their finger-nails grow as a proof that they never condescend to do any kind of manual work. Indian devotees have been known to stand with an uplifted arm till their nails have grown as long as their fingers; and Nebuchadnezzar's nails grew like birds' claws during the seven years he was out grazing. These are things we read about, but we do not often see anything of the kind. Here the growth is actually shown—one nail at least four or five inches long, and curled round like a horn. A similar phenomenon is shown in the skeleton of a rabbit, in which, however, it is a tooth that has thus grown unchecked.

The rat, it has been said, is bound to be continually nibbling and gnawing at something, to keep down the persistent growth of its teeth, or they would be its death. Cases have been known in which they have lost teeth in one jaw, and had therefore nothing for the opposite incisors to work against. The consequence has been that the unchecked tooth has become a tusk, which in one instance was observed to have curled round over the head and caused the death of the animal by piercing the brain. The rabbit in this collection seems to have met with a similar misfortune, only in this case the tusk curled down under the mouth in such a way as to muzzle the unlucky rodent, and by preventing its feeding starved it to death. In one bottle is a kitten born with a hare-lip; in another is a veritable Cyclops—a child with only one eye, and that in the middle of its forehead; and near it is a pig similarly malformed. A pleasanter object to look upon is a small bottle containing a series of tadpoles, showing the creature in every stage of its progress, from the early tadpole to the frog; and there are a great many other objects of minor importance.

In one of the galleries are some deplorable-looking objects in the shape of the skeletons of monkeys that have been badly afflicted with rickets; and among the models in the same gallery are one or two showing the result of that remarkable operation, which is said to have become by no means infrequent—that of covering an artificial nose with a patch of the natural skin brought down from the forehead. The most noteworthy bit of skin in the collection most persons would consider to be that taken from the body of a man named Bishop, and which has been tanned into leather. Says Hamlet:—

> "Imperial Cæsar, dead, and turn'd to clay,
> Might stop a hole to keep the wind away."

Ay; he might, no doubt; and a piece of his skin might make a razor-strop or a pair of dancing-pumps for a ball-room. This particular bit of human leather has attached to it an interest which might have given quite a different turn to the sombre wit of the Prince of Denmark. The man Bishop—whose skeleton, as well as a piece of his skin, is in the museum here—came down to the hospital one day, a good many years ago, with the dead body of a young woman for sale. The experienced eye of the surgeon with whom he had to do saw at a glance that there had been something wrong. He made some excuse for detaining the man until a constable could be fetched, and presently handed him over to safe keeping. The cold-blooded wretch had actually murdered the woman and brought her body down for sale, and that skeleton there has actually hung beneath the gallows.

